# Supplementary material for: Programmed knockout mutation of liver fluke granulin attenuates virulence of infection-induced hepatobiliary morbidity
Source: eLife. 2019 Jan 15;8:e41463. doi: 10.7554/eLife.41463 (PMC6355195; doi:10.7554/eLife.41463)
Supplement: Transparent reporting form [file elife-41463-transrepform.docx]

***eLife’s* transparent reporting form**

We encourage authors to provide detailed information *within their submission* to facilitate the interpretation and replication of experiments. Authors can upload supporting documentation to indicate the use of appropriate reporting guidelines for health-related research (see [EQUATOR Network](http://www.equator-network.org/%20)), life science research (see the [BioSharing Information Resource](https://biosharing.org/)), or the [ARRIVE guidelines](http://www.plosbiology.org/article/info:doi/10.1371/journal.pbio.1000412) for reporting work involving animal research. Where applicable, authors should refer to any relevant reporting standards documents in this form.

If you have any questions, please consult our Journal Policies and/or contact us: [editorial@elifesciences.org](mailto:editorial@elifesciences.org).

**Sample-size estimation**

- You should state whether an appropriate sample size was computed when the study was being designed
- You should state the statistical method of sample size computation and any required assumptions
- If no explicit power analysis was used, you should describe how you decided what sample (replicate) size (number) to use

Please outline where this information can be found within the submission (e.g., sections or figure legends), or explain why this information doesn’t apply to your submission:

Sample sizes were not computed for the study design. However, for studies with liver fluke infection of hamsters, 10 hamsters were included in each treatment group, of five were euthanized at each of two time points, i.e. at 14 days and at 60 days after infection. The pathophysiological changes in each hamster were investigated, including in stained thin-sections of the liver from each hamster in each group. We consider that this sample size was appropriate given that this number has been used in other published reports for experimental studies on pathophysiology of liver fluke infections of hamsters, e.g. Gouveia MJ et al 2017 *Carcinogenesis* 38(9), 929-937. doi: 10.1093/carcin/bgx042.

**Replicates**

- You should report how often each experiment was performed
- You should include a definition of biological versus technical replication
- The data obtained should be provided and sufficient information should be provided to indicate the number of independent biological and/or technical replicates
- If you encountered any outliers, you should describe how these were handled
- Criteria for exclusion/inclusion of data should be clearly stated
- High-throughput sequence data should be uploaded before submission, with a private link for reviewers provided (these are available from both GEO and ArrayExpress)

Please outline where this information can be found within the submission (e.g., sections or figure legends), or explain why this information doesn’t apply to your submission:

Three or more biological replicates of each experiment were performed. We included a definition of biological versus technical replicates in the Materials & Methods section, as follows: ‘These biological replicates represented parallel measurements of biologically discrete samples in order to capture any random biological variation. Technical replicates were undertaken as well; these represented repeated measurements of the same sample undertaken as independent measurements of the random noise associated with the investigator, equipment or protocol’.

High throughput sequence data are available as indicated in the Results section at NCBI, National Library of Medicine:

‘…sequence reads are available at GenBank Bioproject PRJNA385864, Biosample SAMN07287348, SRA study SRP110673, accessions SRR5764463-5764618 and SRR8187484-SRR8187487, at <https://www.ncbi.nlm.nih.gov/Traces/study/?acc=SRP110673>, Bioproject, [www.ncbi.nlm.nih.gov/bioproject/PRJNA385864](http://www.ncbi.nlm.nih.gov/bioproject/PRJNA385864) ‘.

**Statistical reporting**

- Statistical analysis methods should be described and justified
- Raw data should be presented in figures whenever informative to do so (typically when N per group is less than 10)
- For each experiment, you should identify the statistical tests used, exact values of N, definitions of center, methods of multiple test correction, and dispersion and precision measures (e.g., mean, median, SD, SEM, confidence intervals; and, for the major substantive results, a measure of effect size (e.g., Pearson's r, Cohen's d)
- Report exact p-values wherever possible alongside the summary statistics and 95% confidence intervals. These should be reported for all key questions and not only when the p-value is less than 0.05.

Please outline where this information can be found within the submission (e.g., sections or figure legends), or explain why this information doesn’t apply to your submission:

These details have been provided in the Results, including in figure legends, for example, Figure 3 legend, ‘Epithelium width/hyperplasia (green bracket) was quantified using ImageJ and plotted as the mean ± SD of five biological replicates (hamsters) from each of group and time point (14 and 60 days). Significant differences were apparent when compared to the uninfected group using the two-way ANOVA with Holm-Sidak multiple comparison test: ***P* ≤ 0.01 and *****P* ≤ 0.0001, and wild-type compared to Δ*Ov-grn-1*, #*P* ≤ 0.05 and ####*P* ≤ 0.0001’.

Raw data are provided in Figure 5, which deals with levels of transcripts in each of 25 individual adult worms derived from gene edited larval flukes.

(For large datasets, or papers with a very large number of statistical tests, you may upload a single table file with tests, Ns, etc., with reference to sections in the manuscript.)

**Group allocation**

- Indicate how samples were allocated into experimental groups (in the case of clinical studies, please specify allocation to treatment method); if randomization was used, please also state if restricted randomization was applied
- Indicate if masking was used during group allocation, data collection and/or data analysis

Please outline where this information can be found within the submission (e.g., sections or figure legends), or explain why this information doesn’t apply to your submission:

Allocation of samples: Following purchase of the laboratory rodents from the vendor, the hamsters were assigned at random to the three treatment groups, 1) control; 2) infection with wild type liver flukes; and 3) infection with gene-edited liver flukes.

**Additional data files (“source data”)**

- We encourage you to upload relevant additional data files, such as numerical data that are represented as a graph in a figure, or as a summary table
- Where provided, these should be in the most useful format, and they can be uploaded as “Source data” files linked to a main figure or table
- Include model definition files including the full list of parameters used
- Include code used for data analysis (e.g., R, MatLab)
- Avoid stating that data files are “available upon request”

Please indicate the figures or tables for which source data files have been provided:

None provided.
